# SCSPOD14, a South China Sea physical oceanographic dataset derived from *in situ* measurements during 1919–2014

**DOI:** 10.1038/sdata.2016.29

**Published:** 2016-04-26

**Authors:** Lili Zeng, Dongxiao Wang, Ju Chen, Weiqiang Wang, Rongyu Chen

**Affiliations:** 1 State Key Laboratory of Tropical Oceanography, South China Sea Institute of Oceanology, Chinese Academy of Sciences, Guangzhou 510301, China

**Keywords:** Hydrology, Physical oceanography

## Abstract

In addition to the oceanographic data available for the South China Sea (SCS) from the World Ocean Database (WOD) and Array for Real-time Geostrophic Oceanography (Argo) floats, a suite of observations has been made by the South China Sea Institute of Oceanology (SCSIO) starting from the 1970s. Here, we assemble a SCS Physical Oceanographic Dataset (SCSPOD14) based on 51,392 validated temperature and salinity profiles collected from these three datasets for the period 1919–2014. A gridded dataset of climatological monthly mean temperature, salinity, and mixed and isothermal layer depth derived from an objective analysis of profiles is also presented. Comparisons with the World Ocean Atlas (WOA) and IFREMER/LOS Mixed Layer Depth Climatology confirm the reliability of the new dataset. This unique dataset offers an invaluable baseline perspective on the thermodynamic processes, spatial and temporal variability of water masses, and basin-scale and mesoscale oceanic structures in the SCS. We anticipate improvements and regular updates to this product as more observations become available from existing and future *in situ* networks.

## Background & Summary

Much of the major progress in marine science has been closely related to the development of *in situ* observations. A lack of observational data can thus seriously hamper the advancement of oceanography^1^. The World Ocean Database (WOD) Project, established by the Intergovernmental Oceanographic Commission (IOC), represents the world’s largest collection of freely available international ocean profile data^2^. The WOD was first released in 1994, and is updated approximately every four years with the most recent WOD13 released in 2013 (refs 3,4). The World Ocean Atlas (WOA) series is a set of gridded, climatological, objectively analyzed fields at standard depth levels produced from the WOD^5,6^. As part of the successful implementation of the international Array for Real-time Geostrophic Oceanography (Argo) project, a multitude of profiling floats have been deployed since 2000 to build a real-time monitoring system for the upper and middle layers of the world’s oceans^7^. The Argo floats provide a valuable monitoring tool to enhance our understanding of the mixed layer and vertical temperature and salinity structures^8^. They allow us, for the first time, to continuously monitor ocean temperature, salinity, and velocity with all real-time data relayed and made publicly available within hours after collection^9^.

As the largest marginal sea in the tropics, the South China Sea (SCS) exerts a significant influence on climate change, and plays a vital role in water mass and energy exchange between the Pacific and Indian Oceans^10^. As part of the Indo-Pacific warm pool, the SCS is also an important component of the Asian monsoon system. It is subject to strong forcing by alternating northeast and southwest monsoon winds, frequent typhoons, seasonal intrusions of the Kuroshio Current, and fresh water inflow from rivers^11,12^. However, our present knowledge of the evolution of the SCS has been limited by sparse and intermittent observational data. As the only research institute focusing on the ocean science of the SCS in China, the South China Sea Institute of Oceanology (SCSIO), Chinese Academy of Sciences (CAS), has made ongoing contributions to oceanographic surveys. Between 1971 and 2014, the SCSIO performed 203 research cruises, involving multiple generations of hydrographers.

The motivation of this study is to collect all available *in situ* observations up to 2014 and assemble the most comprehensive physical oceanographic database for the SCS to date. SCSIO measurements between 1971 and 2014 are incorporated into the dataset, alongside WOD09 and Argo float profiles. After rigorous quality control, temperature and salinity (referred to as T/S hereafter) profiles were used to generate a gridded dataset with a horizontal resolution of 0.25° at 57 standard vertical levels (SCSPOD14 hereafter).

As detailed knowledge of the stratification in the upper waters of the SCS is very sensitive to the number of observations, the mixed layer depth (MLD), isothermal layer depth (ILD), and barrier layer thickness (BLT) were also calculated and included in this SCSPOD14 dataset. As shown in previous studies, the surface layer is especially important for marine science. It records past winter mixing events and the subsequent onset of spring re-stratification, as well as traces of all physical processes occurring in the upper ocean^13^. Surface properties also determine the heat content and mechanical inertia of the layer that directly interacts with the atmosphere^14^. It is therefore hoped that the SCSPOD14 dataset will prove useful in further studies of these scientific issues.

## Methods

### Data collection

The data collected within the SCS (100–125°E, 0–25°N) were taken from three datasets: WOD09, Argo and SCSIO. In the SCS, WOD09 has 39,673 T/S profiles from February 1907 to January 2009, including 21,125 Ocean Station Data (OSD) profiles, 15,857 conductivity–temperature–depth (CTD) profiles, and 2,961 Profiling Float (PFL) profiles. Note that there were only 7 records in 1907, followed by a gap, and measurements did not start again until 1919. In this study, these few records in 1907 are not considered. Therefore, the start time of our dataset is 1919. As for Argo data, 10,140 profiles obtained after January 17 2009 were considered for inclusion in the dataset. Note that Argo float data already present in WOD09 were not considered again.

In addition to the measurements from WOD09 and Argo, the SCSIO provided 11,774 profiles. The SCSIO performed 203 research cruises between 1971 and 2014. Table 1 (available online only) lists all SCSIO cruises and gives the number of selected T/S profiles from each. For example, the SCSIO has organized and carried out 17 Nansha comprehensive investigation cruises since the 1980s. To understand the physical, chemical, and biological aspects of the northern SCS, an annual open cruise has been conducted during late summer by the SCSIO since 2004 (refs 15,16). The original SCSIO shipboard hydrographic data consisted mainly of OSD profiles before 1983, and were obtained with a Neil Brown Mark 3 CTD between 1983 and 2002, and by Sea-Bird Electronics CTD (SBE CTD; e.g., SBE 25, SBE 911, SBE 917, and SBE-911/917 plus) instruments after 2002. The CTD was tank-calibrated for temperature and pressure before and after each cruise. Modern CTD profiles are considered accurate to approximately 0.002 °C in temperature, 0.005 psu in salinity and <0.5% of full-scale pressure in depth^17^. Besides the above major instruments, some complementary measurements were taken by expendable CTD profiler (XCTD) after 2013 under poor sea conditions. For each scientific voyage, all measurements were qualitatively checked, including a duplicate check for similar position and time, stability check for vertical records, and deviation check which is benchmarked against climatic range^10,18–20^.

### Quality control of the observations

**Figure 1:**
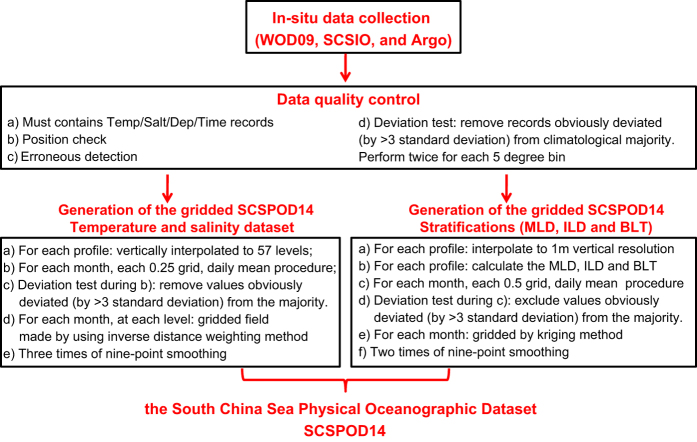
Flow schematic. Flowchart summarizing the main procedures for generating the SCSPOD14 dataset.

At the data collection stage, there are 61,580 records in total. Diverse data sources can lead to variations in the quality and sources of error. For this reason, it was necessary to rigorously control data quality. The main technical processes involved in generating the SCSPOD14 dataset are shown in the flow chart in Fig. 1.

Firstly, valid depth, temperature, and salinity measurements must be included in each observation together with an observation date^21^ (at least year and month). About 1.5% of the measurements that failed this requirement were discarded. Secondly, a careful position check eliminated about 4.4% duplicate stations, those with incorrect location information, stations on land, and those located in shallow water (<15 m). Thirdly, erroneous records (e.g., temperature higher than 8 °C below 1,000 m or salinity lower than 30 psu below 100 m, accounting for about 1.4% of the data) and those with exceptionally sparse vertical resolution (accounting for about 1.1% of the data) were also removed. Further quality control methods, such as deviation test, removing records that obviously deviated (by >3 standard deviation) from climatological majority, were developed and applied, resulting in about 8.1% of the stations being excluded from entering the database^22,23^. This quality-control process reduced the dataset to a total of 51,392 SCS profiles.

### Selected temperature and salinity profiles

**Figure 2:**
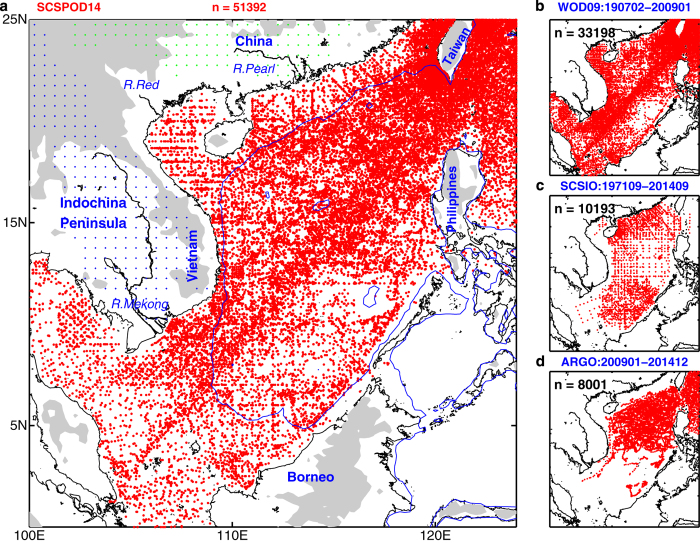
Hydrographic stations. Geographical distribution of hydrographic (temperature and salinity) stations in the South China Sea: (**a**) the merged dataset (SCSPOD14), (**b**) the World Ocean Database 2009 (WOD09), (**c**) South China Sea Institute of Oceanology (SCSIO), and (**d**) Argo profiling floats. The number of profiles (**N**) is shown at the top of each panel.

After the data quality control procedure, 51,392 instantaneous T/S profiles with observations at various levels were selected (Fig. 2a), of which 33,203 were profiles archived in the WOD09 from February 1907 to January 2009; 10,193 were measurements taken by the SCSIO from September 1971 to September 2014; and 8,001 were from Argo profiling floats from January 2009 to December 2013 that are not included in WOD09.

**Figure 3:**
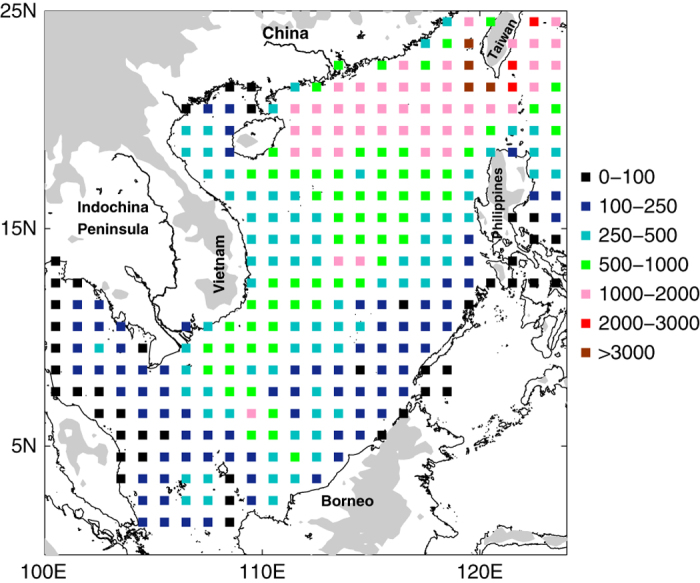
Measurement density. Geographical distribution of station numbers within 1° squares.

The spatial sampling of WOD09 is best in the northeastern and western SCS basin, while the data coverage in the southeastern and northwestern parts of the basin is relatively poor (Fig. 2b). The locations of the hydrographic data acquired by the SCSIO with good quality flags are shown in Fig. 2c. The SCSIO dataset compensates for the lack of measurements in the southern basin to a great extent and further enriches the observations in the northern part. In 2006, Argo floats began to enter the SCS, although these records were very limited until 2008. Figure 2d depicts the spatial coverage of recent profiles from Argo floats that are not included in the WOD09. Argo floats are mainly located in the northern basin, and are associated with surface current patterns (Fig. 2d). The distribution of station numbers in each 0.5° bin further shows the density of samples (Fig. 3). In the northern regions, the combined contributions of the WOD09, SCSIO, and Argo data accounts for the highest measurement density. Although the SCSIO tried to compensate for the sparse observations in the southern basin, the sample density is still relatively low in the southeastern basin.

**Figure 4:**
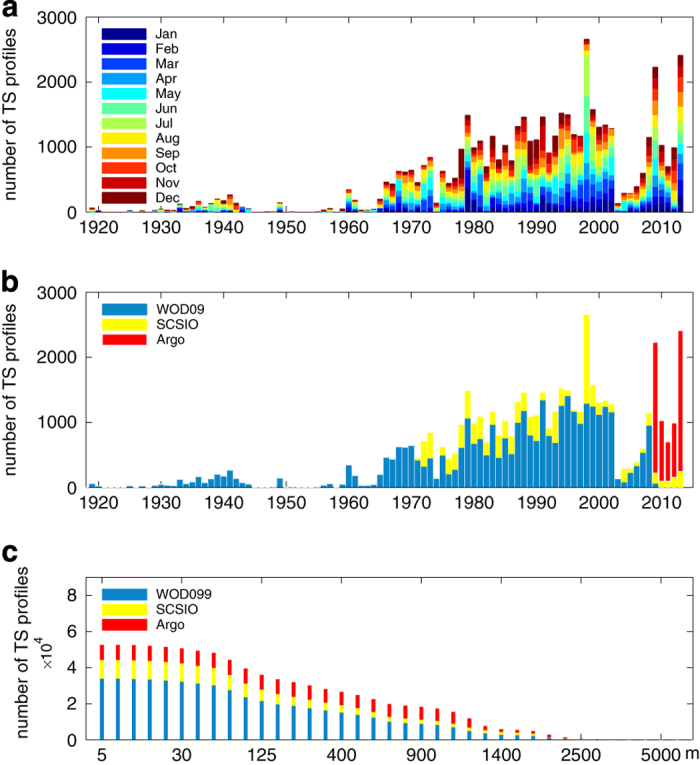
Statistics of hydrographic stations. (**a**) Temporal distribution of the number of T/S profiles of SCSPOD14 in each month; (**b**) temporal distribution of the number of T/S profiles available from WOD09, SCSIO, and Argo; (**c**) distribution of number of T/S profiles available from WOD09, SCSIO, and Argo data at vertical levels.

From the temporal distribution of the records it is clear that measurements in the SCS are relatively scarce in WOD09 prior to 1960 (Fig. 4a and b). The number of data records steadily increases after 1965, reaching its maximum in 1998. After 1970, the number of SCSIO observations was greatly expanded, and complement the WOD09 (Fig. 4b). The distribution of T/S profiles at different depths is shown in Fig. 4c. Most data were collected within the upper 1,000 m of the water column, and there are very limited records below 1,500 m.

### Definitions of upper layer stratification

**Figure 5:**
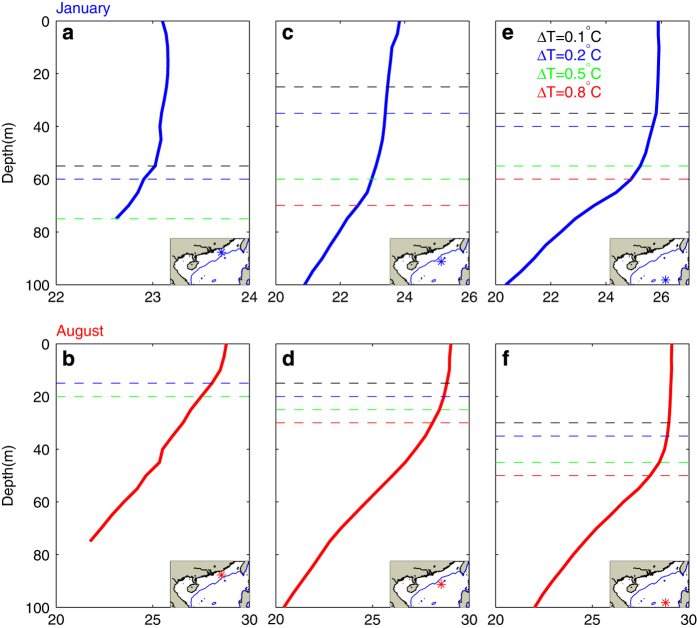
Chosen of temperature threshold. Typical temperature curves of stations located in (**a**,**b**) areas shallower than 200 m, (**c**,**d**) areas just over 200 m, and (**e**,**f**) deep water. Dashed lines indicate the ILD identified by four different temperature thresholds. Upper and lower panels are for January and August, respectively. Locations of the selected stations are shown in the insets.

The analysis of individual profiles allows a more detailed and realistic investigation of physical characteristics, and thus gives more confidence in the description of processes involved^24^. The most widely favored and simplest scheme for finding the ILD is the threshold method^13^. Various temperature thresholds, such as 0.1 °C (ref. 25), 0.2 °C (refs 14,26,27), 0.5 °C (refs 28–30), 0.8  °C (refs 24,31,32) and 1 °C (ref. 33) have been used in the literature. The choice of threshold has an important influence on thermodynamic studies^29^. In this study, after examining numerous profiles, we decided to use different thresholds for shallow and deep-water areas in the SCS. The ILD is defined as the depth where the temperature decreases by 0.8 °C for deep water (deeper than 200 m) and by 0.2 °C for shallow water (less than 200 m) from the temperature at the reference level. The reference depth is taken as 10 m to avoid the diurnal variability in the top few meters of the ocean^14^. The choice of the threshold is based on an inspection of representative samples. Figure 5 shows three typical temperature profiles and four ILD identified by four different thresholds (0.1, 0.2, 0.5, and 0.8 °C). When detecting vertical stratification, the characteristics of shallow-water temperature are distinct from those of deep water, but there is little difference in the temperature profiles in waters just exceeding and much deeper than 200 m. Obviously, ILDs calculated with 0.5 and 0.8 °C thresholds are too large for shallow water regions. Thresholds of 0.1 and 0.2 °C show similar depths in winter, but the 0.1 °C threshold yields ILD that is slightly too shallow in winter, and so 0.2 °C is chosen for the shallow region threshold (Fig. 5a,b). On the other hand, thresholds of 0.1 and 0.2 °C give ILDs that are too shallow for deep-water regions. A comparison of the 0.5 and 0.8 °C values shows that 0.5 °C may give an ILD located in the relatively homogeneous isothermal layer, especially in winter. Comparatively speaking, 0.8 °C is a generally good estimator to distinguish the isothermal and rapidly changing thermocline layers (Fig. 5c–f).

Following previous studies, the MLD is defined where the potential density has increased from its value at the reference level by a given threshold^24,25,27,30–32^. This increment of density is determined by the coefficient of thermal expansion, which is calculated as a function of temperature threshold and salinity at the reference level. The BLT is defined as the difference between ILD and MLD. Each profile was interpolated by a piecewise cubic spline method to obtain 1 m vertical resolution and then to detect the MLD, ILD, and BLT. Sensitivity tests showed that different vertical interpolation methods do not change the stratification depths significantly.

### Generation of the gridded data

The gridded field was produced in three steps. In the first step, each profile was vertically interpolated to the 57 standard depths used in WOA13 (0, 5, 10, 15, 20, 25, 30, 35, 40, 45, 50, 55, 60, 65, 70, 75, 80, 85, 90, 95, 100, 125, 150, 175, 200, 225, 250, 275, 300, 325, 350, 375, 400, 425, 450, 475, 500, 550, 600, 650, 700, 750, 800, 850, 900, 950, 1,000, 1,050, 1,100, 1,150, 1,200, 1,250, 1,300, 1,350, 1,400, 1,450, and 1,500 m). Cubic spline interpolation was used to obtain the values at these new standard layer depths for Argo floats and profiles available at previous standard depths. Linear interpolation was used for SCSIO measurements with higher vertical resolution of 1 m or less. In the second step, we binned the data by month, and then produced a 0.25° field by gridding all temperature and salinity data using an inverse distance weighting method at each vertical standard layer. In this step, to further filter out high frequency variations, such as internal solitary waves in the SCS^34^, the daily mean procedure is executed at each grid before obtaining the gridded value. Given that there are limited observations around some grid points or some standard layers, a variable search radius was adopted; at each grid point, the search radius was gradually increased until it contained at least five historical observations. A standard deviation test was performed during the gridding process, so that the selected profiles were discarded if they deviated from the bin-averaged values by more than three times the standard deviation. In the third step, to partially reduce noise in the observations, three passes of a nine-point smoothing filter were performed after the gridding process at standard levels for monthly compositing periods for the SCS. The two-dimensional nine-point smoothing uses 50% self-weight and 50% adjacent weight from the eight neighboring observation values^14^. Such a weighting scheme enhances the quality and continuity of the climatology compared with a simple linear average approach^35^.

The MLD, ILD and BLT fields estimated from individual profiles were then averaged in 0.5°×0.5° boxes for each month regardless of the year of observation. To eliminate any bias in sampling density, a variable scan radius was chosen so that each average is based on at least 10 samples. If an estimate from an individual profile deviated from the grid mean by more than three times the standard deviation, it was excluded. The monthly gridded MLD, ILD, and BLT fields were then generated using a two-dimensional Kriging method and smoothed by two passes of the nine-point filter. Kriging is an optimal interpolation that generates the best linear unbiased estimate at each location. It is based on regression against observed values of surrounding samples and weighted according to spatial covariance values^36^. The methodology is based on the assumption that irregularly placed MLD/ILD/BLT values are treated regionally. The regionalized MLD/ILD/BLT can then be considered as intermediate between a truly random variable and a completely deterministic variable^37^.

## Data Records

The gridded SCSPOD14 contains two zipped folders from the figshare records (Data Citation 1). The temperature and salinity dataset is available as a zipped folder called Gridded_Temp&Salt_SCSPOD14.zip. The gridded mixed and isothermal layer depth dataset is available in another zipped folder, Gridded_Stratifications_SCSPOD14.zip. Both folders contain asci data files, binary data files (combined with.ctl and.gs files from which GRADS software can read data and output figures) and Matlab data files (.mat) that can be read directly by Matlab software. Additional material such as figures and a README with a detailed description of the content are also available in Data Citation 1.

## Technical Validation

### T/S scatter diagrams

**Figure 6:**
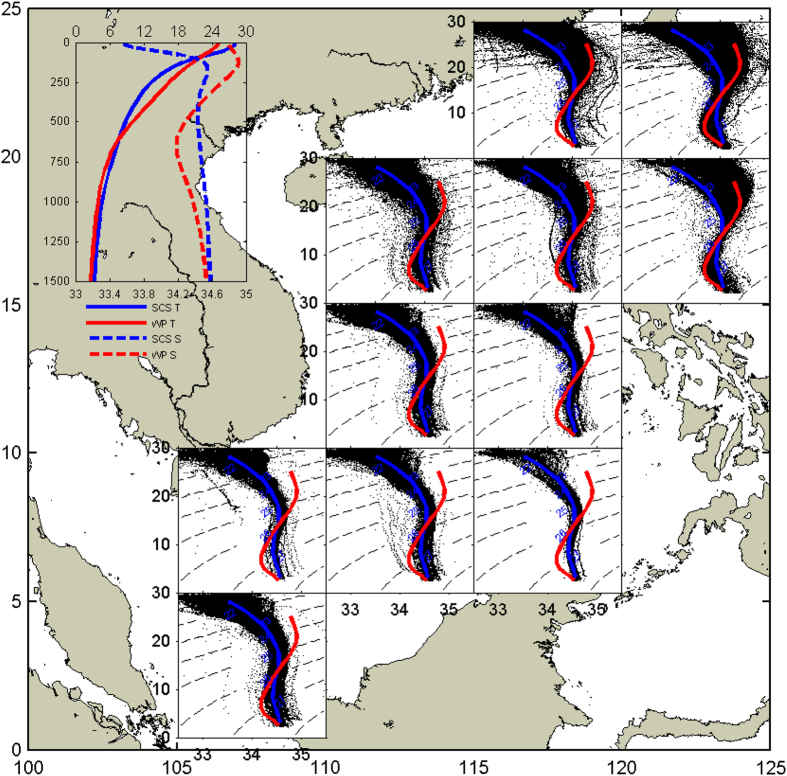
T/S scatter diagrams. Temperature/salinity (T/S) scatter diagrams and average curves for eleven 5°×5° subareas. The red and blue curves in each box are typical T/S curves for the SCS (110–120°E, 5–20°N) and WP (125–180°E, 15–35°N) water masses, respectively. The top left panel shows T and S as functions of depth for these two water masses.

The water properties and exchanges between the SCS and the West Pacific (WP) through the Luzon Strait have been widely documented^38–40^. To verify the general characteristics of the data after quality control, Fig. 6 shows T/S diagrams for every 5° bin in the SCS. The red and blue curves in each box are typical T/S curves for water masses in the SCS (110–120°E, 5–20°N) and WP (125–180°E, 15–35°N), respectively, obtained from the WOA13. A prominent feature is that WP water is warmer and saltier than SCS water, but fresher than intermediate water. Consistent with early studies, the intrusion of WP water is much stronger in the northeastern region of the SCS. As progresses southwestward, most data points fall between the SCS and WP water mass curves. Water masses west of 115°E and south of 15°N exhibit distinct characteristics of the SCS water mass.

### Temperature and salinity: comparison with AMSR-E SST and WOA13 data

To evaluate the SCSPOD14, data inter-comparisons are conducted with the monthly climatological SST measured by microwave sensors from the Advanced Microwave Scanning Radiometer for Earth Observing System (AMSR-E^41^), and monthly climatological temperature and salinity data from the WOA13. The AMSR-E SST is used in the comparison because of the high accuracy of satellite observations.

**Figure 7:**
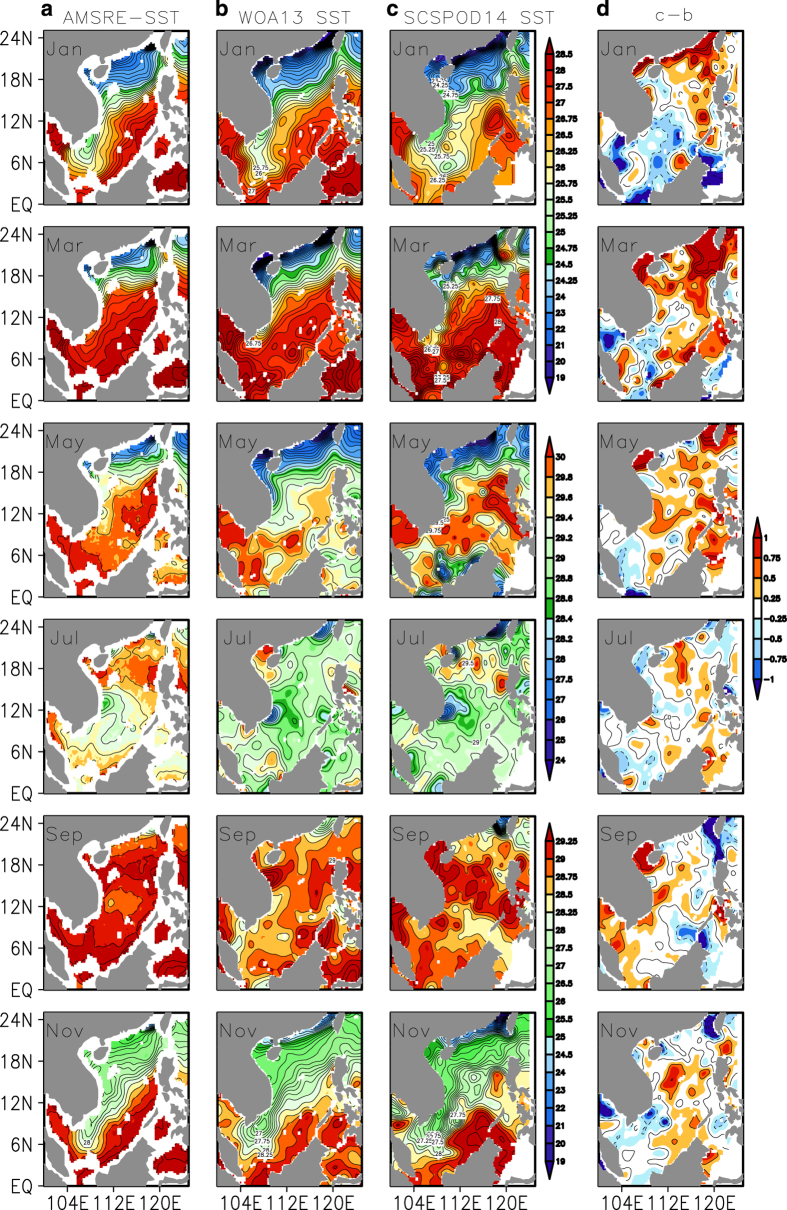
Validation of the SST of SCSPOD14. Bimonthly distributions of the climatological (**a**) AMSR-E SST, (**b**) WOA13 SST, (**c**) SCSPOD14 SST, and (**d**) SST differences between (**c**) and (**b**). Contour intervals for SST and SST differences are 0.25 °C.

Bimonthly distributions of AMSR-E SST, WOA13 SST, and SCSPOD14 SST are shown in Fig. 7a–c. Both WOA13 SST and SCSPOD14 SST depict the seasonal variability of SST in the SCS. Compared with the satellite AMSR-E SST, WOA13 and SCSPOD14 have better spatial coverage in shallow waters. Both WOA13 and SCSPOD14 SST are lower than the satellite AMSR-E SST in continental slope areas. In general, SCSPOD14 SST is higher than WOA13 SST in the northern region, but lower in the southern region. The most significant quantitative differences between these two datasets occur in the northeastern corner in March, where the difference is larger than 0.5 °C (Fig. 7d). The distinct spatial differences between these two datasets occur in the summer monsoon season (e.g., May and July) as shown in Fig. 7b and c. In May, the SCSPOD14 SST field is much closer to that of AMSR-E SST, while the WOA13 SST is much cooler, especially in the central and eastern basin. The difference between SCSPOD14 and WOA13 SST is positive over almost the entire basin. In July, WOA13 SST was still lower than AMSR-E and SCSPOD14 in the northern basin. Unlike their differences in the northern SCS, WOA13 and SCSPOD14 are relatively consistent in the central and southern basin. Both WOA13 and SCSPOD14 show a low temperature core caused by upwelling around 12°N, slightly south of the position of this feature in AMSR-E. In the southern basin, the values in WOA13 and SCSPOD14 are both lower than in AMSR-E. The difference between SCSPOD14 and WOA13 SST is relatively small in July compared with other months. By September, the characteristics of WOA13 and SCSPOD14 SST both differ to some extent from the AMSR-E measurements. In contrast to earlier months, from September the SCSPOD14 SST is cooler than WOA13 SST in continental slope areas, particularly around Taiwan where their difference is about –0.5 °C

**Figure 8:**
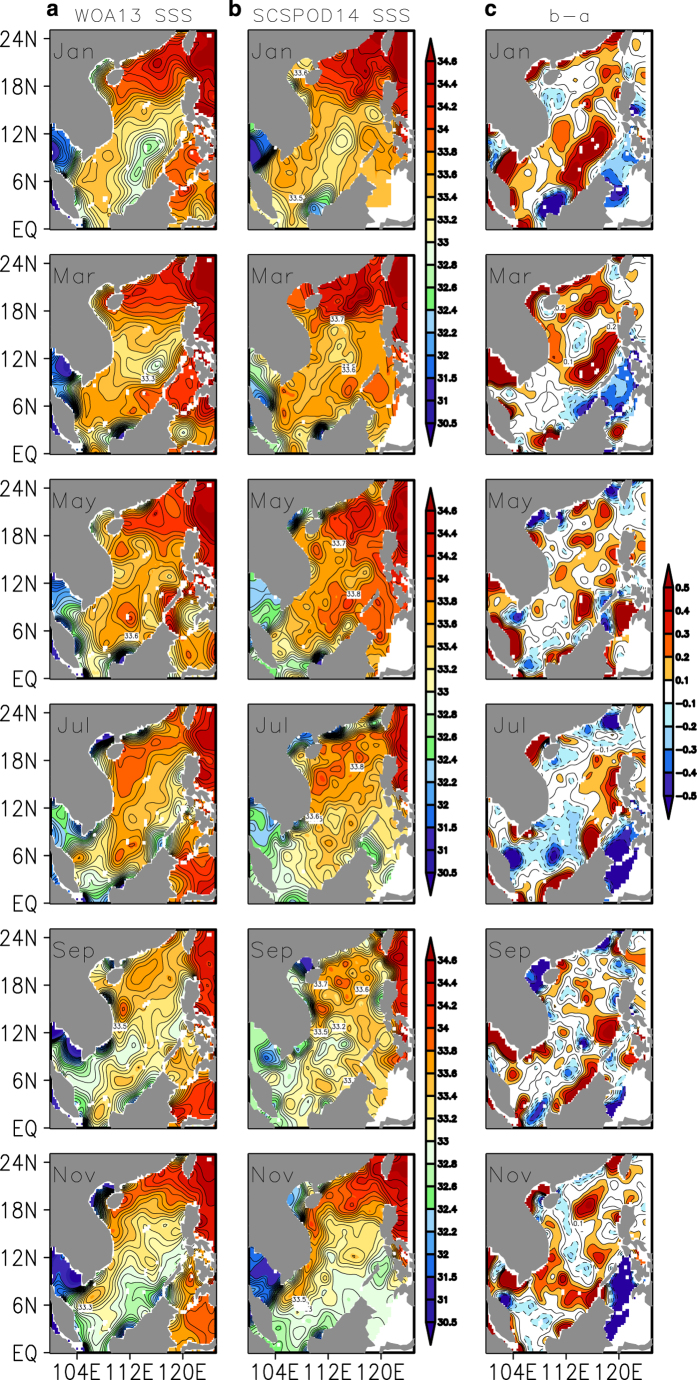
Validation of the SSS of SCSPOD14. Bimonthly distributions of the climatological (**a**) WOA13 SSS, (**b**) SCSPOD14 SSS, and (**c**) SSS differences between (**b**) and (**a**). Contour intervals for SSS and SSS differences are 0.2 psu and 0.1 psu, respectively.

**Figure 9:**
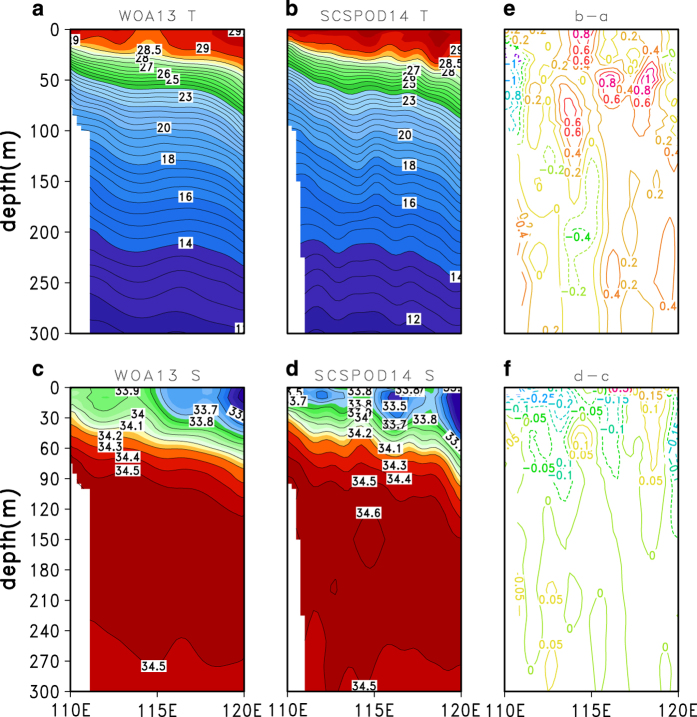
Validation of the temperature and salinity of SCSPOD14. Temperature along the 18°N transect from 0 to 300 m in July from (**a**) WOA13 and (**b**) SCSPOD14; and salinity from (**c**) WOA13 and (**d**) SCSPOD14. (**e**) Temperature difference between (**b**) and (**a**), and (**f**) salinity difference between (**d**) and (**c**). Contour intervals for temperature and salinity are 0.5 °C and 0.1 psu, respectively.

Due to the limitations of satellite salinity data and their poor accuracy in the SCS, this study only shows the comparison between WOA13 and SCSPOD14 SSS. The results indicate that SSS from the WOA13 and SCSPOD14 have similar spatial patterns, with a high-salinity band that spreads from southwest of Taiwan to east of Hainan Island, and forms a high-salinity core in the central northern SCS in winter. In summer and autumn, SSS is lower in the basin with obvious low-salinity zones located near the Mekong River Estuary where intense runoff produces freshwater lenses, and along the east boundary of the SCS where there is abundant precipitation. As shown in Fig. 8a and b, differences in SSS between the datasets are smaller than for SST. The major differences between SCSPOD14 and WOA13 SSS occur in the southeastern basin (Fig. 8c). As described in the data collection section, the difference in observation numbers of the two datasets is also the greatest in this region. The additional measurements in SCSPOD14 might lead to the difference in SSS. Another discrepancy occurs around 18°N where SCSPOD14 also contains many measurements. We believe that more observations will change the salinity dataset. Vertical distributions of temperature and salinity are given in Fig. 9a–d, in addition to SST and SSS. The transect along 18°N in July is selected due to the large differences that occur in that region (Figs 7d and 8c). Located between the tropics and subtropics, this transect is important for studying meridional ocean transport in the SCS. The SCSIO open cruises in the northern SCS have surveyed the 18°N transect on a regular basis since 2004 and obtained multi-year measurements, greatly enriching the observations of this region^10^. Figure 8 shows that the main differences are in the upper ocean, rather than in the subsurface and intermediate waters (not shown). There is a warmer (around 0.5 °C) and fresher (around –0.05 psu) mixed layer in the SCSPOD14 along 18°N compared with WOA13 observations (Fig. 9e and f). The contrast is more obvious in the western basin than in the eastern basin. In contrast to the WOA13, a secondary high salinity core around 150 m appears in the SCSOD14.

### Upper ocean stratification: comparison with IFREMER/LOS MLD climatology

The differences seen in the T/S profiles in the upper ocean can affect the calculation of stratification in the SCS. Therefore, new upper layer stratification data is also provided in the SCSPOD14 dataset. Because of the various definitions and calculations of upper layer stratification, there are no direct reference data for comparison. Here, we only evaluate the SCSPOD14 stratifications by comparison with global climatological data, the IFREMER/LOS Mixed Layer Depth Climatology^14^ produced by de Boyer Montégut *et al.* Their two kinds of MLD calculation are used: MLD-T02 uses the temperature threshold of 0.2 °C, while MLD-R003 uses a density criterion of 0.03 kg m^−3^ difference from the reference depths as 10 m.

**Figure 10:**
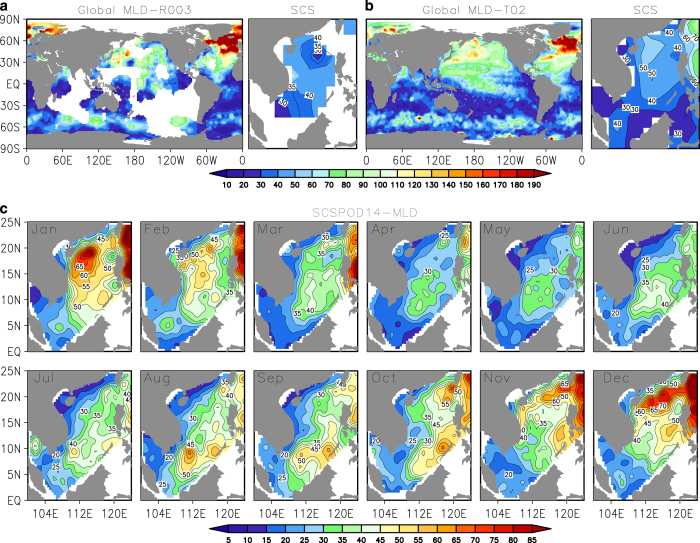
Validation of the MLD of SCSPOD14. The IFREMER/LOS MLD Climatology distribution in January, (**a**) MLD-R003, (**b**) MLD-T02. (**c**) Monthly distributions of the SCSPOD14 MLD with 5 m contour interval.

From Fig. 10a,b, both MLD-R003 and MLD-T02 in the IFREMER/LOS climatology cannot provide high-resolution MLD data in the SCS. It is also clear that the coverage of MLD-R003, which depends on the density and thus requires both the temperature and salinity profiles, is more insufficient compared to MLD-T02, which depends only on temperature profiles. The difference between the two MLD calculation methods is significant over the SCS, which further provides evidence for the existence of the barrier layer in the SCS. This emphasizes the utility of our new MLD, ILD, and BLT calculations in the SCSPOD14 dataset.

The monthly MLD distribution is shown in Fig. 10c. As a result of numerous *in situ* measurements, the SCSPOD14 MLD depicts the seasonal change in detail. During winter, the deep tongue extending southwestward from the Luzon Strait along the continental slope in the northern basin exceeds 50 m to the west of 119°E. Zones of deep ILD and MLD form in this region, associated with predominantly northeasterly winds, surface cooling, and convergence. Our maps of the climatology also clearly show a shallow center around the West Luzon Eddy northwest of the Philippines. During summer, a deep core occurs in the southeastern basin and spreads southwestward, reaching a maximum depth of 45 m. Our climatology also clearly shows a band of shallow MLD, connecting two shallow centers, one around the West Luzon Eddy northwest of the Philippines during winter, and another located in the West Vietnam Cyclonic Eddy east of Vietnam during summer. Note that only the figures and MLD validation are provided here; figures and related documents for the ILD and BLT are provided in Data Citation 1.

These comparisons of the gridded SCSPOD14 data with WOA13 and IFREMER/LOS MLD climatology clearly show the value of SCSPOD14 for the study of oceanic structure, water masses, and the assessment of the accuracy of numerical simulations. We are confident that this dataset will be a useful complement to other gridded products. This work also highlights the need for more observations in the southeastern basin to fill spatial gaps in the dataset.

## Additional Information

**How to cite this article:** Zeng, L. *et al.* SCSPOD14, a South China Sea physical oceanographic dataset derived from *in situ* measurements during 1919–2014. *Sci. Data* 3:160029 doi: 10.1038/sdata.2016.29 (2016).

## Supplementary Material



## Figures and Tables

**Table 1:** SCSIO research cruises

**No**	**R/V**	**Area**	**T/S**	**No**	**R/V**	**Area**	**T/S**	**No**	**R/V**	**Area**	**T/S**	**No**	**R/V**	**Area**	**T/S**	**No**	**R/V**	**Area**	**T/S**
1	197109	PRD	39	42	197711	NWSCS	26	83	198106	NSCS	47	124	198706	NSCS	44	165	199510	NESCS	11
2	197112	PRD	65	43	197712	NWSCS	29	84	198107	NWSCS	11	125	198708	NWSCS	43	166	199511	NWSCS	26
3	197203	NSCS	169	44	197801	NWSCS	26	85	198108	NSCS	47	126	198710	NSCS	53	167	199608	NSCS	25
4	197204	NWSCS	52	45	197802	NWSCS	29	86	198109	NSCS	36	127	198712	NSCS	58	168	199702	NSCS	25
5	197205	NSCS	182	46	197803	NWSCS	29	87	198110	NSCS	47	128	198745	SSCS	82	169	199708	NSCS	38
6	197207	NESCS	39	47	197804	NWSCS	26	88	198111	NSCS	43	129	198802	NSCS	43	170	199711	SSCS	140
7	197208	NWSCS	77	48	197805	NWSCS	29	89	198112	NSCS	47	130	198804	NSCS	42	171	199802	NSCS	25
8	197210	NWSCS	78	49	197806	NWSCS	29	90	198204	NSCS	47	131	198806	NSCS	42	172	199804	SCS	496
9	197303	NWSCS	234	50	197807	NSCS	47	91	198206	NSCS	47	132	198808	SSCS	92	173	199806	SCS	796
10	197305	NWSCS	130	51	197808	NSCS	47	92	198208	NSCS	47	133	198810	NWSCS	25	174	199807	SCS	50
11	197306	NWSCS	104	52	197809	NSCS	41	93	198210	NSCS	42	134	198812	NSCS	47	175	199809	NWSCS	13
12	197308	PRD	26	53	197810	NSCS	34	94	198212	NSCS	47	135	198902	NSCS	38	176	199902	NSCS	25
13	197309	NWSCS	104	54	197811	NSCS	42	95	198302	NSCS	47	136	198905	NSCS	38	177	199904	SSCS	36
14	197504	NWSCS	16	55	197812	NSCS	42	96	198304	NSCS	47	137	198908	NSCS	41	178	199907	SSCS	241
15	197505	NWSCS	16	56	197901	NSCS	43	97	198306	NWSCS	21	138	198911	NSCS	38	179	199908	NSCS	38
16	197506	NWSCS	14	57	197902	NSCS	47	98	198308	NSCS	47	139	198912	SSCS	152	180	200008	SCS	189
17	197507	NWSCS	14	58	197903	NSCS	47	99	198309	CSCS	42	140	199002	NSCS	41	181	200103	NESCS	97
18	197508	NWSCS	14	59	197904	NSCS	42	100	198310	NESCS	16	141	199003	SCS	22	182	200205	SSCS	124
19	197509	NWSCS	16	60	197905	NSCS	47	101	198312	NSCS	48	142	199005	NSCS	41	183	200405	SSCS	139
20	197510	NWSCS	16	61	197906	NSCS	47	102	198402	NSCS	47	143	199008	NSCS	41	184	200409	NSCS	71
21	197511	NWSCS	16	62	197907	NSCS	47	103	198404	C-NSCS	82	144	199011	NSCS	39	185	200509	NSCS	54
22	197512	NWSCS	12	63	197908	NSCS	46	104	198406	NSCS	47	145	199056	SSCS	234	186	200609	NSCS	61
23	197601	NWSCS	11	64	197909	NSCS	47	105	198407	C-NSCS	89	146	199101	NSCS	41	187	200709	NSCS	61
24	197602	NWSCS	16	65	197910	NSCS	45	106	198410	NSCS	42	147	199105	NSCS	41	188	200806	NESCS	58
25	197603	NWSCS	16	66	197911	NSCS	21	107	198412	C-NSCS	89	148	199108	NSCS	38	189	200807	NESCS	58
26	197604	NWSCS	13	67	197912	NSCS	17	108	198502	NSCS	47	149	199111	NWSCS	25	190	200808	NSCS	80
27	197605	NWSCS	29	68	198001	NSCS	47	109	198504	NSCS	47	150	199203	NSCS	37	191	200905	SCS	104
28	197606	NWSCS	28	69	198002	NWSCS	22	110	198506	NSCS	47	151	199205	NSCS	38	192	200909	NSCS	54
29	197607	NWSCS	29	70	198003	NESCS	11	111	198508	NSCS	42	152	199208	NWSCS	25	193	201009	NSCS	29
30	197608	NWSCS	29	71	198004	NESCS	25	112	198510	NSCS	40	153	199211	NSCS	38	194	201011	SCS	67
31	197609	NWSCS	27	72	198005	NSCS	47	113	198512	NSCS	43	154	199302	NSCS	41	195	201109	NSCS	58
32	197610	NWSCS	29	73	198006	NSCS	47	114	198556	SSCS	64	155	199305	SSCS	164	196	201111	SCS	37
33	197612	NWSCS	25	74	198007	NESCS	11	115	198602	NSCS	40	156	199308	NWSCS	25	197	201208	SCS	75
34	197702	NWSCS	22	75	198008	NSCS	47	116	198604	NSCS	42	157	199311	NSCS	41	198	201210	C-NSCS	51
35	197704	NWSCS	29	76	198009	NSCS	27	117	198606	NSCS	47	158	199312	SSCS	189	199	201212	NWSCS	32
36	197705	NWSCS	29	77	198010	NSCS	41	118	198608	NSCS	47	159	199402	NESCS	16	200	201308	WSCS	143
37	197706	NWSCS	29	78	198011	NSCS	42	119	198610	NSCS	47	160	199403	SSCS	34	201	201310	NSCS	90
38	197707	NWSCS	30	79	198012	NESCS	22	120	198612	NSCS	47	161	199408	NSCS	41	202	201403	NSCS	101
39	197708	NWSCS	29	80	198102	NSCS	43	121	198645	SSCS	75	162	199409	SSCS	165	203	201409	WSCS	107
40	197709	NWSCS	28	81	198104	NSCS	42	122	198702	NSCS	47	163	199502	NSCS	41				
41	197710	NWSCS	29	82	198105	NWSCS	30	123	198704	NSCS	58	164	199505	NSCS	41				
The 203 SCSIO research cruises from 1971 to 2014 showing cruise number, date, area covered, and number of T/S profiles. Note that R/V corresponds to year and month in the format yyyymm. Abbreviations: PRD, Pearl River Delta; NSCS, Northern South China Sea; SSCS, Southern South China Sea; WSCS, Western South China Sea; NWSCS, Northwestern South China Sea; NESCS, Northeastern South China Sea; C-NSCS, Central and Northern South China Sea.																			
